# A new protein evaluation system for horse feed from literature data*

**DOI:** 10.1017/jns.2014.66

**Published:** 2015-02-04

**Authors:** Annette Zeyner, Susanne Kirchhof, A. Susenbeth, K.-H. Südekum, Ellen Kienzle

**Affiliations:** 1 Department of Animal Nutrition, Martin-Luther-University Halle-Wittenberg, Halle (Saale), Germany; 2 Institute of Animal Nutrition, University of Hohenheim, Stuttgart-Hohenheim, Germany; 3 Institute of Animal Nutrition and Physiology, Christian-Albrechts-University, Kiel, Germany; 4 Institute of Animal Science, Rheinische Friedrich-Wilhelms-University, Bonn, Germany; 5 Chair for Animal Nutrition and Dietetics, Ludwig-Maximilians-University Munich, Munich, Germany

**Keywords:** Pre-caecal digestibility, Protein evaluation, Amino acids, Horse feed

## Abstract

Few data on apparent pre-caecal digestibility (APCD) of crude protein (CP) and particularly amino acids (AA) are available from studies with horses. Protein bound in cell walls (i.e. neutral detergent insoluble CP (NDICP)) is unlikely to be decomposed by digestive enzymes in the small intestine. In contrast the corresponding analytical fraction of neutral detergent soluble CP (NDSCP) (NDSCP = CP−NDICP) is likely to be available for auto-enzymatic digestion. A literature analysis on the relationship between NDICP/NDSCP and pre-caecal indigestible/digestible CP was carried out. There was a strong positive relationship between NDICP and pre-caecal indigestible CP, which suggests that NDICP can be used to estimate the part of protein that is not available for digestion in the small intestine. There was also a correlation between NDSCP and pre-caecal digestible protein. The slope of the linear regression line between NDICP and pre-caecal digestible CP was 0·9, suggesting an APCD of NDSCP of 90 %. Thus pre-caecal digestible CP may be predicted by multiplying NDSCP by 0·9. Because the literature identifies a similar AA profile in NDICP and NDSCP within a given feed the presented concept may preliminarily be transferred to AA. The proposed system can at any time be adapted to the scientific progress without altering its structure. Such adaptations would be necessary particularly when new knowledge exist on the distribution of AA onto NDICP/NDSCP, the APCD of individual AA from NDSCP, and the impact of feed processing and chewing on particle sizes and protein digestibility.

For the horse, the exclusive source of amino acids (AA) are derived from protein digested in the small intestine^(^^1^^–^^3^^)^. Ideally, protein evaluation of feeds for horses should be based on small intestinal protein and AA digestibility. There are, however, not enough data on the apparent pre-caecal digestibility (APCD) of crude protein (CP) in horses to establish such a system based on experimentally determined APCD of CP from different feeds. Protein bound in plant cell walls is unlikely to be available for pre-caecal digestion in monogastric animals. This protein fraction can be analysed as neutral detergent insoluble CP (NDICP) by the ‘Cornell Net Carbohydrate and Protein System’ for cattle^(^^4^^)^. NDICP may be used to estimate the soluble part of the protein (neutral detergent soluble CP (NDSCP)) as the difference between CP and NDICP. NDSCP is equivalent to protein of the cell content that can potentially be decomposed by digestive enzymes in the small intestine after being released from plant structures by the chewing process.

AA profiles of both, NDICP and the corresponding fraction NDSCP, appear to be similar within a given feed^(^^5^^,^^6^^)^. Thus based on the present knowledge it seems to be justified to transfer the AA profile of the whole feedstuff to both protein fractions NDSCP and NDICP. The aim of the present study was to investigate on the basis of a literature analysis whether a concept of protein evaluation on the basis of soluble and insoluble protein might be applicable to horse feed.

## Material and methods

The following literature was used to investigate whether the NDSCP/NDICP concept can be applied to estimate apparent pre-caecal digestible CP (APCDCP) and apparent pre-caecal digestible AA (APCDAA) in horse feed: NDICP and NDSCP in feedstuffs^(^^7^^–^^9^^)^; pre-caecal digestible CP from studies with horses^(^^10^^–^^19^^)^; pre-caecal digestible AA from experiments with horses^(^^20^^,^^21^^)^.

For statistical analyses, the relationship between the intakes of (i) NDICP and pre-caecal indigestible CP, and (ii) NDSCP and pre-caecal digestible CP was determined by means of linear regression analysis (SPSS 18.0 for Windows, Chicago, IL, USA). For this, literature that reported the ingested quantities, not only the concentrations of NDICP, NDSCP and the pre-caecal digestible and indigestible parts of CP in the feed were used. Furthermore, data from experiments where the feed intake was considerably below the maintenance level were not included because results were presumed to be compromised by endogenous losses. According to these both preconditions, a total of nine digestibility trials were identified to be suitable for statistical analysis^(^^10^^,^^11,^^16^^,^^17^^)^. Data from these studies were based on digestibility trials with meadow grass, roughages (grass hay and lucerne hay), cereal grains (oats, barley and maize) and rations containing hay and cereal grains in ratios being 1:0, 3:2 and 1:4.

## Results

**Fig. 1.**
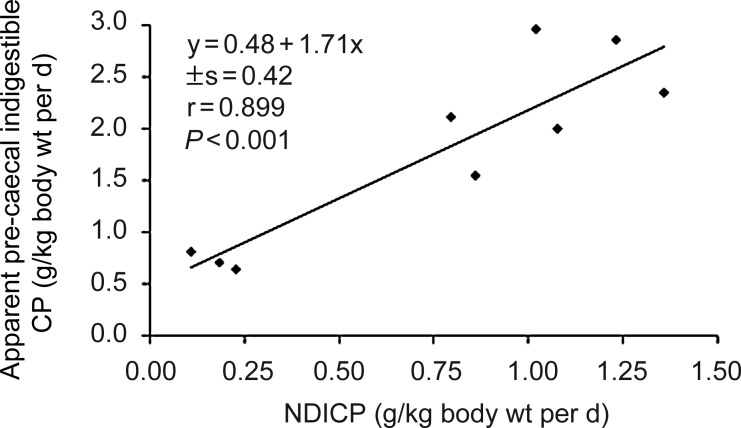
Relationship between the intake of neutral detergent insoluble crude protein (NDICP) and experimentally determined apparent pre-caecal indigestible CP in different feedstuffs and rations for horses (*n* 9^(10,11,16,17)^).

The intake of NDICP was positively correlated with the intake of experimentally determined pre-caecal indigestible CP (Fig. 1) over the earlier described wide range of horse feed. There was also a strong positive correlation between the intake of NDSCP and pre-caecal digestible CP (*r* 0·895; *P* < 0·001). The slope of the corresponding linear regression line was 0·9 suggesting an APCD of NDSCP of 90 %.

## Discussion

The results of the literature analysis indicate that the analytical fraction of NDICP in the feed can be used to estimate the parts of CP, which are not available to the horse, and, vice versa, those which are available for digestion in the small intestine (equation 1): (1)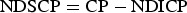 Assuming an APCD of NDSCP of 90 % estimated by the slope of the regression line between intakes of NDSCP and APCDCP, the content of APCDCP in any feedstuff in question can be calculated as given in equation (2):
(2)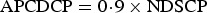 The concept might even be extended to AA, provided the AA pattern of the feed is known. For this, equation (3) can be used to determine APCDAA: (3)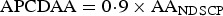 where AA_NDSCP_ represents the content of the AA in question in the soluble protein fraction assuming that it is nearly the same as in the total CP^(^^5^^,^^6^^)^. Until further evidence is available it is especially important to characterize feedstuffs for horses with different characteristics (brood mares, growing horses, high-performance horses and geriatric horses) particularly according to the feedstuffs' content of APCD lysine and threonine followed by methionine and cysteine^(^^22^^,^^23^^)^.

When the method is applied to three fairly typical horse feeds such as oats, fresh grass and grass hay (1st cut), e.g. CP and NDICP contents of 123, 144, 107 and 17, 21, 44 g/kg DM, respectively, the corresponding content of APCDCP is 106, 123 and 63 g/kg DM. This looks like a promising start for an improved protein evaluation system based on NDICP in horses. The database is small, the concept is still rather hypothetical, and it needs further development. For instance, the AA distribution into NDICP and NDSCP needs to be specified, and the absorbability of individual AA from the soluble part of CP identified and considered in the system. Furthermore, in silages ammonia must be taken into account (NDSCP = CP − NDICP − 6·25 NH_3_-N). Other non-protein N compounds may lead to an overestimation of APCDCP. Free AA are likely even more available than AA from NDSCP, it is instead recommended to assume an APCD of 100 %. NDICP describes an important chemical and physical barrier to protein digestion. There may be mechanical barriers to protein digestion in the small intestine such as plant structures. This was demonstrated for starch digestion^(^^24^^)^ and may also be true for protein digestion. Sample preparation by grinding in the laboratory will destroy most of the mechanical barriers to protein digestion but chewing by the horse may not. Processing of feed may also induce Maillard-reactions leading to the production of protein compounds which may not be digestible in the small intestine but will not appear in NDICP. Nevertheless, the use of this preliminary system in practice is likely to give important impetus to the research on protein and AA availability in horses. Therefore the German Committee for Requirement Standards of the Society of Nutrition Physiology^(^^25^^)^ decided to use the method as a future protein evaluation system in Germany.
